# Relieved Low Back Pain after Total Hip Arthroplasty in Patients with Both Hip Osteoarthritis and Lumbar Degenerative Disease

**DOI:** 10.1111/os.13135

**Published:** 2021-10-04

**Authors:** Tian‐fei Ran, Song Ke, Jie Li, Ming‐rui Lyu, Yuan‐yuan Zhou, Rui Zhang, Xin Song, Min Wang

**Affiliations:** ^1^ Department of Orthopaedics Xinqiao Hospital, Amy Medical University (Third Military Medical University) Chongqing China; ^2^ Department of Social Medicine and Health Service Management Army Medical University Chongqing China

**Keywords:** Hip osteoarthritis, Hip‐spine syndrome, Low back pain, Spinal degenerative diseases, Total hip arthroplasty

## Abstract

**Objective:**

To investigate the relief of low back pain after hip arthroplasty in patients with hip joint and spinal degenerative diseases, and to discuss the effects of unilateral and bilateral hip surgery on the relief of low back pain.

**Methods:**

In this retrospective study, we followed 153 patients (69 males and 84 females, age: 43–88 years) who had undergone total hip arthroplasty (THA) *via* a posterolateral approach and also suffered from lumbar degenerative diseases in the period of 2009 to 2019. The inclusion criteria were: (i) patients who had been diagnosed with severe hip degenerative disease and also been diagnosed with lumbar degenerative disease; (ii) patients who had undergone THA surgery; and (iii) patients who were retrospectively recruited. The exclusion criteria were: (i) patients who had undergone lumbar fusion or internal fixation surgery; or (ii) patients who had vascular claudication, history of major trauma, diabetic polyneuropathy, lumbar and pelvic infections, tumor diseases; (iii) or patients who had undergone THA because of femoral neck fracture or ankylosing spondylitis. The improvement of hip joint function and the relief of low back pain (LBP) were studied, and the effect of unilateral and bilateral THA on the relief of LBP were discussed. Hip pain and function were evaluated by the Harris Hip Score (HHS), LBP was evaluated by Visual Analog Scale (VAS), and lumbar function was evaluated by the Japanese Orthopaedic Association (JOA) scoring system.

**Results:**

The average follow‐up time was 44.3 months (24–108 months). All patients recovered smoothly without complications. The LBP VAS of 153 patients decreased from 4.13 ± 1.37 preoperatively to 1.90 ± 1.44 postoperatively. The average HHS increased from 45.33 ± 13.23 preoperatively to 86.44 ± 7.59 postoperatively at the latest follow‐up. According to Japanese Orthopaedic Association scoring system, the proportion of patients with good response to treatment in these 153 patients reached 93.46%. LBP VAS decreased from 4.18 ± 1.38 preoperatively to 1.95 ± 1.49 postoperatively in unilateral group and from 3.94 ± 1.32 preoperatively to 1.73 ± 1.23 postoperatively in bilateral group, respectively. There were only nine patients with persistent or aggravated LBP after operation. Among them, six patients underwent subsequent lumbar surgery (five patients had pain relieved after reoperation and one patient had not) and the other three patients chose conservative treatment for pain.

**Conclusion:**

THA can relieve LBP while relieving hip pain and restoring hip function in patients with both hip and lumbar degenerative disease, thus possibly avoiding further spinal surgery.

## Graphical abstract

After THA, LBP in some patients will be effectively relieved, thus avoiding further spinal surgery. Therefore, the results of this study highlight the need to treat hip osteoarthritis first when patients have both hip disease (hip osteoarthritis, osteonecrosis of the femoral head, or both) and lumbar degenerative disease.

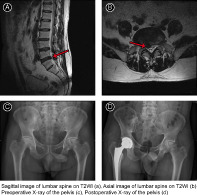

## Introduction

According to statistics, one in two patients with symptomatic hip joint degeneration suffers from low back pain (LBP)^1^. As aging intensifies, more and more patients have back pain and lower limb discomfort caused by hip joint disease and lumbar degenerative disease^2^, ^3^, ^4^, ^5^, ^6^. It is reported that among patients undergoing total hip arthroplasty (THA), the proportion of patients suffering from different degrees of low back pain ranged from 21.2% to 60.4%^7^, ^8^, ^9^, ^10^.

In people with pathological changes of the hips, pain distributions can include the groin, lateral hip, and posterior pelvis. However, the most common site of pain is in the buttocks, followed by thighs and groins^11^. Usually, degenerative diseases of the lumbar spine cause radiation pain and numbness in the lower limbs. And imaging data play an important role in the differential diagnosis of these two diseases. Patients with hip discomfort can be examined with X‐ray at the first visit by identifying pathological changes of hip osteoarthritis, which include osteophyte hyperplasia at the edge of the femoral head and acetabular fossa, subchondral bone cyst formation, and joint space stenosis^12^. Lumbar degenerative diseases can be evaluated by lumbar X‐ray combined with magnetic resonance imaging (MRI).

However, some of the symptoms of these two diseases (hip joint disease and lumbar degenerative disease) are the same, such as lower limb pain, pain around the knee joint, abnormal gait, and LBP^13^, ^14^, ^15^. Therefore, it is difficult to identify which disease is causing the patient's current symptoms, and even more difficult to diagnose when both diseases occur in the same patient. At the same time, in the treatment of those patients, the problem that which surgery (hip surgery or lumbar surgery) should be the first occurs. Failure to recognize this close pathological relationship between the spine and hip joint may delay treatment and lead to unsatisfactory surgical outcomes of the hip or spine^16^, ^17^. Therefore, joint surgeons and spinal surgeons need to work closely together to treat such patients.

Offierski and MacNab^18^ first described this association of hip osteoarthritis (OA) and spinal disorders as hip‐spine syndrome (HSS) in 1983. They classified this syndrome into three groups, as follows: (i) simple HSS defined by changes between hip joint and spinal pathology, but with the obvious pain and disability originating from only one source; (ii) secondary HSS defined as aggravated spine syndrome caused by the hip deformity, e.g., restriction of hip motion in patients with advanced OA may cause a positive sagittal balance deformity and consequent LBP; and (iii) complex HSS defined by degenerative changes of the hip and lumbar spine that both contribute to the pain of patients and overlap with one another, with the source of the pain and the cause of the disability unable to be clearly identified.

Previous studies indicated that low back pain in patients with hip osteoarthritis was relieved after THA and emphasized the need to treat hip osteoarthritis primarily. Ben‐Gallim *et al*.^19^ published a study on the intervention of patients with LBP and hip OA. In this study, the pain and function scores of 25 patients were evaluated before and after THA. All results showed a statistically significant improvement after THA, and reassessment of back pain following hip arthroplasty showed possible diminished symptoms and obviated the need for spinal treatment. Moreover, Piazzolla *et al*.^20^ reported that patients with concomitant unilateral HOA and LBP having a marked anteverted FNA in the arthritis were observed to have both hip pain and LBP relieved and obtain a change in spinopelvic parameters after THA.

However other studies have shown that patients with a history of THA have more back pain after low back surgery than patients with no history of total hip replacement^21^. Up to now, the operation sequence has been controversial^3^, ^5^, ^6^. If a proper surgical sequence is found and accepted by both joint and spinal surgeons, preoperative counseling will be easier, patients will get better treatment, and even the associated treatment burden will be reduced.

Some scholars have reported the relief of low back pain in patients with hip osteoarthritis after total hip arthroplasty. However, knowledge about the relief of LBP in patients with degenerative changes in both hip and lumbar who first underwent THA surgery is limited and there is no study yet to analyze the effect of unilateral and bilateral THA on the relief of LBP. Therefore, the purpose of this study was: (i) to follow up a group of patients with hip joint disease and lumbar degenerative disease; (ii) to observe their hip joint pain relief and function recovery as well as LBP relief after THA; and (iii) to explore the effect of unilateral THA and bilateral THA on such patients.

## Methods and Patients

### Patients

#### Inclusion Criteria

The inclusion criteria were: (i) patients who had been diagnosed with severe hip degenerative disease and also been diagnosed with lumbar degenerative disease (hip joint lesions include osteoarthritis, necrosis of the femoral head, or both; lumbar degenerative diseases include lumbar disc herniation [LDH], degenerative lumbar spondylolisthesis [DLS], degenerative scoliosis [DS], and lumbar spinal stenosis [LSS]); (ii) patients who had undergone THA surgery; (iii) Harris Hip Score, Japanese Orthopaedic Association (JOA) score, and Visual Analog Scale were compared; (iv) patients who were retrospectively recruited.

#### Exclusion Criteria

The exclusion criteria were: (i) patients who had undergone lumbar fusion or internal fixation surgery; or (ii) patients who had vascular claudication, history of major trauma, diabetic polyneuropathy, lumbar and pelvic infections, tumor diseases; (iii) or patients who had undergone THA because of femoral neck fracture or ankylosing spondylitis. Patients who met any of the exclusion criteria were excluded from the study.

**Fig. 1:**
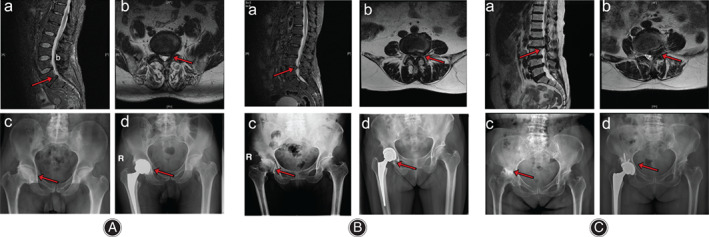
Typical images of three patients with both lumbar degenerative disease and hip disease who underwent total hip arthroplasty. (A) A 56‐year‐old man who had lumbar disc herniation (L_5_S_1_) and right osteonecrosis of the femoral head. (B) A 67‐year‐old woman who had lumbar disc herniation (L_4‐5_) and right osteonecrosis of the femoral head. (C) A 70‐year‐old man who had lumbar disc herniation (L_2‐3_) and right osteonecrosis of the femoral head.

The subjects of this study were patients who received THA treatment in the orthopaedic department of our hospital from 2010 to 2019. Figure 1 shows three typical patients who have degenerative lumbar and hip diseases and underwent THA.

This study was a retrospective study. All subjects received and accepted informed consent before participating in the study, which was approved by the institutional review committee of our hospital.

### Diagnosis

Hip joint diseases and lumbar degenerative diseases were diagnosed by experienced joint surgeons through imaging examination including X‐ray, computed tomography (CT), and magnetic resonance imaging (MRI) and also assessed by them in combination with the current symptoms.

### Surgery

The surgical method is standard posterolateral approach for total hip arthroplasty.

#### Anesthesia and Position

Because of spinal disease, all patients underwent THA under intravenous inhalation combined anesthesia. Patients were in the lateral position.

#### Approach and Exposure

All procedures were performed *via* a posterolateral approach. After incision of the skin along the muscle fiber line, the tensor fasciae latae and gluteus maximus were divided, the external rotators and the joint capsular were incised.

#### Resection

Osteotomy along the femoral neck was performed to remove the femoral head, followed by removing the labrum, femoral head ligaments, and other adipose or fibrous tissue. Then, the acetabulum and femoral canal were carefully prepared.

#### Placement of Prosthesis and Reconstruction

**Fig. 2:**
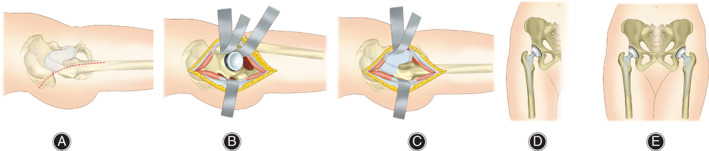
Intraoperative pictures of unilateral and bilateral total hip arthroplasty. (A) Surgical incision of posterolateral approach. (B) Exposure of the capsule through posterolateral approach. (C) Installation the components of the prosthesis. (D) Unilateral total hip arthroplasty. (E) Bilateral total hip arthroplasty.

The acetabular cup and femoral stem were implanted by ensuring optimal position and orientation. Then, the hip joint was reset, and the incision was closed (Fig. 2).

### Clinical Evaluation

We collected and analyzed the preoperative and postoperative Harris Hip Score (HHS), Japanese Orthopaedic Association (JOA) score, and low back pain (LBP) Visual Analog Scale (VAS) score of these patients. At the same time, in order to analyze the effect of unilateral and bilateral THA surgery on the relief of LBP, we divided the patients into unilateral THA surgery group and bilateral THA surgery group, and compared the difference between the two groups in follow‐up results.

### Data Collection

The in‐patient data of the patients such as height, weight, gender, age, phone number, and operation time were obtained from the hospital database. HHS was routinely evaluated before THA. For patients with lumbar diseases, JOA and LBP VAS were also evaluated. We also collected the results of the latest follow‐up. In the event that the patient underwent subsequent lumbar surgery because of the aggravation of lumbar pain or had reoperation for other reasons, only the score before reoperation was included in statistics.

### Scale of Clinical Evaluation

#### Harris Hip Score (HHS)

HHS was used to evaluate the hip joint function and pain relief before and after operation. The HHS score system mainly includes four aspects: pain, function, absence of deformity, and range of motion. The scoring standard took 100 points as the highest score (best possible outcome)^22^.

#### Japanese Orthopaedic Association (JOA) Scoring System and Recovery Rate (RR)

JOA (0–29) scoring system was used to evaluate lumbar function before and after operation. It consisted of three parts: symptoms (9 points); signs including straight‐leg raise (6 points); and seven activities of daily living (ADL, 14 points)^23^. The score 29 represents normal function and 0 represents the worst.

RR was used to evaluate the effective rate of low back pain relief. According to Recovery Rate (%) = (postoperative score − preoperative score)/(29 – preoperative score) × 100% in JOA scoring system, RR was calculated and divided into four groups: cured (RR = 100%); markedly effective (60% ≦ RR ≦ 99%); effective (25% ≦ RR ≦ 60%); and ineffective (RR ≦ 25%)^24^.

#### Visual Analog Scale (VAS)

VAS was used to evaluate the pain of the low back pre and post operation. It ranges from 0 to 10, where 0 represents no pain and 10 the worst possible pain^25^.

### Statistical Analysis

The data was analyzed by a statistical software (Version 25.0, SPSS, IBM, Armonk, NY, USA), and the quantitative data were expressed as mean ± standard deviation (SD). A *t*‐test was used to analyze the preoperative scores and postoperative follow‐up results of the 153 patients. Independent *t*‐test was used to analyze the LBP VAS and HHS of unilateral and bilateral THA groups. A two‐tailed probability level of *P* < 0.05 was selected as the statistically significant level.

## Results

### Basic Patient Data

According to the inclusion criteria and exclusion criteria, finally 160 patients met the criteria, among which seven patients died and were lost to follow‐up, and 153 patients were included in the study. The typical images of the patients were shown in Fig. 1. The average age of male patients was 62.93 (47–80) years and that of female patients was 65.23 (43–88) years at the time of operation. The average follow‐up time was 44.3 months (24–108 months). All patients received unilateral or bilateral THA treatment in the orthopaedic department of our hospital from 2010 to 2019, including 120 cases of unilateral THA and 33 cases of bilateral THA. These operations were performed by three experienced joint surgeons. Demographic data and surgical information are shown in Table 1.

**TABLE 1:** Demographic data of the study

Demographic	Male	Female
Gender (cases [%])	69 (45.1)	84 (54.9)
Age (mean [range], years)	62.93 (47–80)	65.23 (43–88)
BMI (mean [range], kg/m^2^)	24.06 (17.80–33.17)	24.29 (15.39–33.74)

BMI, body mass index.

### Clinical Results

#### Hip and Lumbar Function of Whole Group

**Fig. 3:**
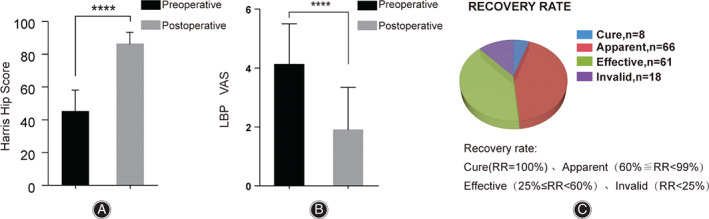
Data on low back pain (LBP) VAS, recovery rate (RR) and hip function of pre and post total hip arthroplasty of those patients with both lumbar degenerative disease and hip joint disease. Based on tests, (A) comparing the data of pre‐hip operation with that of post‐hip operation there was a significant improvement (*P* < 0.0001) in the Harris Hip scores for pain relief and function recovery of hip, and (B) there was a significant decrease (*P* < 0.0001) in the LBP VAS. (C) Based on the calculation of recovery rate (RR) in JOA scoring system, that eight patients were cured, 66 were apparent, 61 were effective, and 10 were invalid. All values are statistically calculated as Mean ± SD. ^****^ *P* < 0.0001.

One hundred and fifty‐three patients had significant relief of low back pain after surgery. The mean LBP VAS decreased from 4.13 ± 1.37 preoperatively to 1.90 ± 1.44 postoperatively (*P* < 0.0001). The average HHS increased from 45.33 ± 13.23 preoperatively to 86.44 ± 7.59 postoperatively (*P* < 0.0001). According to the calculation method of RR in JOA scoring system, 8 cases were cured, 66 markedly effective, 61 effective, and 10 ineffective. The effective rate of low back pain relief was 93.46% (Fig. 3).

#### Hip and Lumbar Function between Unilateral and Bilateral Surgery Groups

**Fig. 4:**
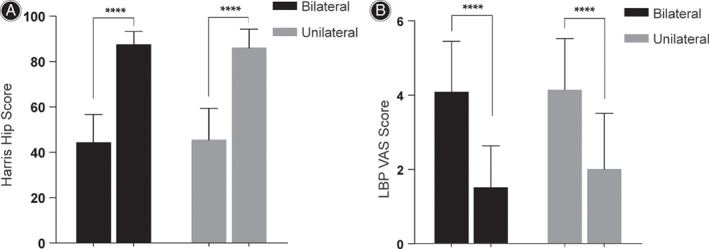
Harris Hip score (HHS) and low back pain (LBP) VAS in bilateral and unilateral pre‐ and post‐total hip arthroplasty. Based on the *t*‐tests, the (A) HHS and (B) LBP VAS for pain relief and function recovery of hip in patients with bilateral and unilateral THA were significantly improved (*P* < 0.0001) after operation. All values are statistically calculated as Mean ± SD.

In our study, 120 patients received unilateral THA and 33 patients received bilateral THA. The follow‐up results of the two groups showed significant improvement: LBP VAS decreased from 4.18 ± 1.38 preoperatively to 1.95 ± 1.49 postoperatively in unilateral group and from 3.94 ± 1.32 preoperatively to 1.73 ± 1.23 postoperatively in bilateral group, respectively (*P* < 0.0001); HHS increased from 45.59 ± 13.59 preoperatively to 86.13 ± 8.07 postoperatively in unilateral group and from 44.39 ± 12.02 preoperatively to 87.58 ± 5.42 postoperatively in bilateral group, respectively (*P* < 0.0001). There was no significant difference in preoperative or postoperative HHS and LBP VAS between unilateral and bilateral groups (*P* > 0.05). However, interestingly, the patients who received unilateral THA had better relief of low back pain and recovery of lumbar function than those who received bilateral THA, and the difference was statistically significant (Fig. 4, Table 2).

**TABLE 2:** Harris hip score and VAS scores of patients who underwent unilateral and bilateral THA

Scores		Bilateral (*n* = 33)	Unilateral (*n* = 120)	*P* value
Harris Hip Score	Preoperative	44.39 ± 12.02	45.59 ± 13.59	0.67
	Postoperative	87.58 ± 5.42	86.13 ± 8.07	0.33
	*P* value	**<0.000**	**<0.000**	
LBP VAS score	Preoperative	3.94 ± 1.32	4.18 ± 1.38	0.37
	Postoperative	1.73 ± 1.23	1.95 ± 1.49	0.43
	*P* value	**<0.000**	**<0.000**	

Bold denotes statistically significant at *P* < 0.05. All values are statistically calculated as Mean ± SD.

### Complications and Reoperation

All patients recovered smoothly without complications. Although most of the patients' pain was relieved or even disappeared, there were nine patients with persistent or aggravated pain after operation. Among them, six patients underwent subsequent lumbar surgery (five patients had pain relieved after reoperation and one patient didn't) and the other three patients chose conservative treatment for pain. Their information is shown in Table 3.

**TABLE 3:** Reoperation data of six patients after THA

Case	Diagnosis	Hip surgery	Spine surgery	Interval (years)	Pain
1	Hip OA + LDH	Unilateral THA	MIS‐TLIF	1	Relief
2	Hip OA + LDH		MIS‐TLIF	1.5	Relief
3	Hip OA + LDH		MIS‐TLIF	1.8	Relief
4	Hip OA + LDH		MIS‐TLIF	2.5	Persistent
5	Hip OA + DLS		LSF	2	Relief
6	Hip OA + LSS		PVP	1	Relief

DLS, degenerative lumbar spondylolisthesis; Hip OA, Hip osteoarthritis; LDH, lumbar disc herniation; LSF, lumbar spinal fusion; LSS, lumbar spinal stenosis; MIS‐TLIF, minimally invasive surgery‐transforaminal lumbar fusion; PVP, percutaneous vertebroplasty; THA, total hip arthroplasty.

## Discussion

### Low Back Pain Eased after Total Hip Arthroplasty

In this retrospective study we followed up 153 patients who were diagnosed with both hip joint disease and lumbar degenerative disease before THA. They all received THA in our hospital from 2010 to 2019. These operations were performed by three experienced joint surgeons. Through our follow‐up, it was found that the symptoms of LBP in these patients eased with the relief of hip pain and improvement of hip function after THA. Their average LBP VAS score decreased from preoperative 4.13 ± 1.37 to postoperative 1.90 ± 1.44 (*P* < 0.0001). At the same time, according to the RR calculation formula of the JOA scoring system, the proportion of patients with good treatment response to LBP reached 93.46%. Our results show that LBP has been relieved after THA, which is consistent with previous studies^7^, ^10^, ^16^. Although most patients' pain was relieved or even disappeared, there were nine cases of persistent or worsening pain postoperatively. Eight of them achieved good results after receiving medications or subsequent lumbar spine surgery (Table 3).

### Low Back Pain was Relieved in Unilateral and Bilateral Groups

To the best of our knowledge, this study is also the first to discuss the effects of unilateral and bilateral THA surgery on patients with both hip and lumbar degenerative diseases. In our study, it was found that the follow‐up results of patients in the unilateral and bilateral surgery groups were both significantly improved. Although there was no significant difference in preoperative and postoperative HHS and LBP VAS between the unilateral surgery group and the bilateral surgery group, it is interesting to find that the LBP VAS of patients who received unilateral THA showed greater change and the difference was statistically significant (Fig. 3, Table 2). This means that patients receiving unilateral THA treatment have better pain relief than patients receiving bilateral THA treatment.

It has been reported that patients receiving bilateral THA had higher hip pain level preoperatively than that in the unilateral group, but there was no difference in the scores between the two groups after 1 year of surgery, which is somewhat not consistent with our preoperative results of HHS^26^. This difference in preoperative scores may be related to our small sample. Since our study is the first to discuss the effects of unilateral and bilateral THA treatment on these patients, it is difficult for us to search out similar studies to compare the results we obtained.

### Mechanisms of Low Back Pain Relief

There are many possible reasons for LBP relief after THA. We believe that the following reasons may be helpful in the interpretation of the follow‐up results obtained in this study.

Firstly, several publications describe that THA is helpful to improve the spinal and pelvic parameters to restore the balance of the spine, which may be one of the mechanisms of LBP relief. As Weng *et al*.^27^ reported that THA was helpful to correct the abnormal arrangement of spine‐pelvis‐leg sagittal position and reduced the comorbid LBP, preoperative and postoperative lateral radiographs in their study showed that the hip flexion of the 69 patients they recruited was significantly reduced and the overall spinal balance was improved; of the 39 patients who reported LBP, 17 reported complete remission and 22 reported significant remission. Another study^20^ reported that patients with concomitant unilateral HOA and LBP who showed a marked anteverted FNA in the arthritis, after THA experienced relief of both hip pain and LBP, and a change in spinopelvic parameters was observed.

Secondly, Chimenti *et al*.^7^ reported that social factors affect the mitigation of LBP. They followed up over 2800 patients undergoing unilateral primary THA. A total of 60.5% (1707/2820) of the patients reported mild or larger LBP preoperatively, and 58.4% (997/1707) of the patients had LBP relieved postoperatively. A comparison between patients with relieved LBP and patients with unrelieved LBP showed that patients with relieved LBP were more likely to have private medical insurance, received a college education or higher education, earnt a household income of more than $45,000, had a lower burden of chronic disease, and suffered from less joint pain. However, the mechanism for the relief of LBP after THA may be more complicated, which requires more in‐depth study and investigation. In particular, the pain relief in the unilateral surgery group is more obvious, which may become a new breakthrough in exploring the relief of low back pain after THA.

### Hip Spine Syndrome: Which Operation First

Offierski and MacNab first described the connection between hip osteoarthritis (OA) and spine disease as hip spine syndrome (HSS) in 1983^18^. Failure to recognize this close pathological relationship between the spine and hip joint may delay treatment and lead to unsatisfactory surgical outcomes of the hip or spine^16^, ^17^. Some patients with hip disease and patients with lumbar degenerative disease may show the same or similar symptoms: hip and lower limb pain, sometimes pain around the knee, gait abnormalities, changes in the sagittal sequence of the lumbar spine, and low back pain symptoms. Many studies reported that the sagittal imbalance of the spine was correlated with low back pain and hip symptoms, and a variety of measurement methods have been used to confirm that. Therefore, the above facts will cause great trouble for joint surgeons to diagnose. What's more, in the treatment of such patients, the problem of the sequence of hip surgery and lumbar spine surgery, which is first, will arise. So far, the sequence of surgeries for patients with hip osteoarthritis and lumbar degenerative disease has been controversial.

### THA First Can Relieve LBP

Previous studies have shown that the surgical sequence may bring different effects to patients^8^, ^15^, ^20^, ^28^. There is no doubt that THA can effectively relieve pain and restore function in patients with advanced hip arthritis^28^, ^29^, ^30^. At the same time, some related studies have shown that patients with coexisting hip and lumbar degenerative diseases have lumbar diseases treated after THA^7^, ^8^, ^20^, and they recommend hip surgery first. Ben‐Gallim *et al*. published a study on the intervention of patients with LBP and hip OA. In this study, the pain and function scores of 25 patients were evaluated before and after THA. All results showed a statistically significant improvement after THA. The authors concluded that THA relieved LBP and recommended hip surgery first^19^. However, a small sample in their study may be considered a defect. In addition, another shortcoming of their study is that the patients they studied did not indicate whether there were pathological changes in the lumbar spine, which is very important for the interpretation of the results. Another study also found that patients with a history of lumbar spine fusion surgery to undergo THA had worse early outcomes and higher rates of complications and reoperation^28^.

Combined with our above follow‐up results, for diagnosis and surgical treatment of patients with hip joint disease and lumbar degenerative disease, we suggest focusing on symptoms. In patients with pathological changes of hips, the most common site of pain is in the buttocks, followed by thighs and groin^11^. However, lumbar degenerative disease can also lead to lower limb pain and dysfunction, which will lead to overlapping symptoms between the hip joint disease and lumbar degenerative disease, and needs further identification^31^, ^32^, ^33^. At this time, detailed physical examination is helpful to the diagnosis of the disease. While patients with hip OA usually have inguinal pain, claudication or hip internal rotation limitation, which can induce lower limb pain during weight‐bearing, hip internal rotation and external rotation, lower limb rolling test^34^, and imaging examination can also help provide further diagnostic information. Hence, patients with hip discomfort can be examined with X‐ray at the first visit by identifying pathological changes of hip osteoarthritis, which include osteophyte hyperplasia at the edge of the femoral head and acetabular fossa, subchondral bone cyst formation, and joint space stenosis^34^. Since the diagnosis of early osteonecrosis can only be achieved by MRI, low back MRI is recommended for patients who are still difficult to diagnose^35^, ^36^. At the same time, it is necessary to identify the lesions around the hip joint, such as acetabular dysplasia, glenoid lip tear, round ligament tear, synovitis, trochanteric bursitis, etc. If the cause of the current symptoms can be determined before the operation, then appropriate treatment can be carried out. However, when there are pathological changes in the hip joint and spine, we recommend THA first. But patients also need to be advised that performing surgery on one anatomical site can relieve the symptoms, but may also exacerbate symptoms in another anatomical site.

The following lumbar degenerative diseases need to be considered separately: lumbar disc herniation leading to nucleus pulposus falling out, cauda equina compression, lumbar spondylolisthesis leading to significant spinal canal stenosis. These patients are advised lumbar surgery first, but they also need to receive THA following the lumbar surgery, because the symptoms caused by hip joint diseases will not be relieved before THA.

### Limitations

This study also has limitations. A main limitation is that we did not analyze the imaging data of spine and pelvis in these patients after THA, which may have helped us to further explore the LBP relief mechanism. The next step of our study plan is to follow them up for a long time to analyze the imaging data of their spine and pelvis. Secondly, this is a single‐center retrospective study, which has its inherent limitations. Thirdly, the sample size of the bilateral THA group is relatively small compared with the unilateral THA group. These limitations should be taken into consideration in interpreting our results and should be addressed in future studies.

## Conclusions

The results of this study highlight the need to treat hip osteoarthritis first when patients have both hip disease (hip osteoarthritis, osteonecrosis of the femoral head, or both) and lumbar degenerative disease. After THA, LBP in some patients will be effectively relieved, thus avoiding further spinal surgery. Of course, for patients with cauda equina compression or severe lumbar lesions, more detailed and prudent surgical planning is needed.
